# Body composition of children with moderate and severe undernutrition and after treatment: a narrative review

**DOI:** 10.1186/s12916-019-1465-8

**Published:** 2019-11-25

**Authors:** Jonathan C. K. Wells

**Affiliations:** Childhood Nutrition Research Centre, Population, Policy and Practice Research and Teaching Department, University College London (UCL) Great Ormond Street Institute of Child Health, 30 Guilford Street, London, WC1N 1EH UK

**Keywords:** Child undernutrition, Undernutrition, Body composition, Wasting, Stunting

## Abstract

**Background:**

Until recently, undernourished children were usually assessed using simple anthropometric measurements, which provide global assessments of nutritional status. There is increasing interest in obtaining more direct data on body composition to assess the effects of undernutrition on fat-free mass (FFM) and its constituents, such as muscle and organs, and on fat mass (FM) and its regional distribution.

**Main text:**

Recent studies show that severe-acute undernutrition, categorised as ‘wasting’, is associated with major deficits in both FFM and FM that may persist in the long-term. Fat distribution appears more central, but this is more associated with the loss of peripheral fat than with the elevation of central fat. Chronic undernutrition, categorised as ‘stunting’, is associated with deficits in FFM and in specific components, such as organ size. However, the magnitude of these deficits is reduced, or – in some cases – disappears, after adjustment for height. This suggests that FFM is largely reduced in proportion to linear growth. Stunted children vary in their FM – in some cases remaining thin throughout childhood, but in other cases developing higher levels of FM. The causes of this heterogeneity remain unclear. Several different pathways may underlie longitudinal associations between early stunting and later body composition. Importantly, recent studies suggest that short children are not at risk of excess fat deposition in the short term when given nutritional supplementation.

**Conclusion:**

The short- and long-term functional significance of FFM and FM for survival, physical capacity and non-communicable disease risk means that both tissues merit further attention in research on child undernutrition.

## Background

Despite substantial progress in prevention and treatment, child undernutrition remains a major global health challenge and still contributes to around half of all deaths in children under 5 years of age [1]. Greater understanding of how undernutrition affects different body components might enable the development of clinical and public health interventions that are more effective in promoting survival and long-term quality of life, through reversing tissue deficits and their functional consequences.

Until recently, undernourished children were usually assessed using simple anthropometric measurements, which provided global assessments of nutritional status. This approach built on conceptual advances made in the 1970s, when the pioneering nutritionist John Waterlow proposed a distinction between ‘chronically’ and ‘acutely’ undernourished children, based on measurements of height or weight [2]. Subsequently, this evolved into the routine practice of categorising children as either ‘stunted’ (defined as having an inadequate height for age z-score, HAZ), or ‘wasted’ (defined as having an inadequate weight for height z-score, WHZ, or mid-upper arm circumference, MUAC). This approach can be further adapted to differentiate between moderate underweight-for-height, versus severe underweight-for-height, or wasting [3].

The nutrition research and practice community has widely adopted these practices and numerous reports now quantify the prevalence of wasted and stunted children [1]. There is growing recognition that such a dichotomous approach is artificial, however, as individual children may be simultaneously wasted and stunted [4] and each condition increases the subsequent risk of the other developing [5]. However, this review maintains this terminology, simply because this is how data have been reported to date.

To understand the physical consequences of undernutrition more fully, the simplest model of body composition divides mass into two components: fat-free mass (FFM) and fat mass (FM) [6]. Each of these components is of interest, as there is limited evidence that each is associated with the likelihood of survival in early life. First, when insufficient dietary protein or amino acids are available, muscle mass may provide critical proteins for immune function. Simple markers of depleted muscle mass have been associated with higher mortality risk [7, 8]. Potential effects of undernutrition on non-muscle components of FFM have received very little attention. It is possible that organs are generically protected at the expense of muscle; however, reductions in thymus size associated with undernutrition have been linked with reduced immune competence [9]. Second, FM provides metabolic precursors and energy for immune function, which has high metabolic costs [10]. Moreover, FM secretes leptin; a ‘gateway’ hormone for immune function [11]. Two studies of children with severe-acute undernutrition have shown that mortality can be predicted by low levels of leptin [12, 13].

In recent decades, researchers have increasingly measured body composition in undernourished children. As increasing numbers of children survive undernutrition in the short-term, new questions are emerging regarding the long-term consequences and effects of treatment [14–16]. The aim of this non-systematic review is, first, to briefly review the body composition analysis methods that are appropriate for use in undernourished children and then to summarise published data on this topic. I will consider how body composition is associated with undernutrition at baseline, how it changes during treatment and the longer-term effects.

### Methodological issues

Although simple anthropometry (body mass index, BMI) is often used as a proxy for body composition, its associations with fatness are generally weak [17]. This approach is also particularly unsuitable for assessing associations between stunting and body composition. When height is incorporated in measurements of both exposure (HAZ) and outcome (BMI), any error in height measurement generates an autocorrelation between short stature and high BMI [18, 19]. Thus, early reports linking stunting with an elevated risk of overweight categorised by BMI [20] should be treated with caution. Direct measurement of adiposity is recommended.

There is no gold standard for body composition assessment – other than cadaver dissection. All in vivo techniques are necessarily imperfect and require assumptions to convert from raw measurements to final body composition values [6]. It is challenging to obtain data from young children – more so if they are unwell. Nonetheless, objective methods have recently been applied to undernourished children, including deuterium dilution (D_2_O) [14, 15], dual-energy X-ray absorptiometry (DXA) [21, 22] or bioelectrical impedance analysis (BIA) [14, 16]. In specialised research centres in high-income countries, it is also possible to obtain accurate information using magnetic resonance imaging (MRI) [23] or multi-component models (MCM) that rely on fewer theoretical assumptions [24].

A related challenge is that, until recently, minimal reference data were available for healthy individuals [25], other than the estimated average tissue masses of the ‘reference child’ of Fomon et al. [26]. Several publications from high-income countries have described the full range of healthy body composition parameters – in some cases for younger age groups, such as birth to 2 years in the US (MCM) [27] and 6 weeks to 5 years in the UK (D_2_O) [28]. However, these have limited relevance to low- and middle-income country (LMIC) settings, where sustained environmental stresses (e.g., constrained food supply, infectious diseases) affect growth of the entire population to some degree. Reference data from birth to 6 months of age were published for Ethiopia (using air displacement plethysmography) [29] and for children aged ≥5 years in India [30, 31]. In the absence of similar data from other LMICs, measuring healthy controls from the same setting remains essential for interpreting data [16], although these controls may also have experienced undernutrition earlier in life.

Body composition is closely associated with body size. When researching the immediate or long-term consequences of undernutrition, it is important to disentangle size variability from that of body composition [25]. Calculating percentage body fat is a flawed approach, as high values might reflect either high FM or low FFM [32]. A more appropriate approach is to adjust tissue masses for height, dividing each of FFM and FM by height-squared. The resulting fat-free mass index (FFMI) and fat mass index (FMI) are both expressed in the same kg/m^2^ units as BMI [33]. Reference data for these outcomes are emerging [28] and height-adjusted outcomes are increasingly reported in child undernutrition research [15, 34, 35].

Methodological issues remain in need of further research. First, for example, if undernutrition coexists with oedema, many of the assumptions used to convert raw data into final body composition values are violated [36]. The data reported below are currently the best available, but may still suffer from limitations, depending on the severity of undernutrition. Second, most data relate only to FFM and FM, and this limits our understanding of the exact nature of FFM depletion and its functional consequences.

### Body composition following severe-acute undernutrition

The major weight loss that characterises wasted children may impact both fat and fat-free tissue. However, the effect of hydration perturbations on measurement techniques makes it difficult to obtain accurate data. In a UK study using MCM, adolescents with the eating disorder anorexia nervosa had 1.4 z-scores lower FM than controls and 1.1 z-scores lower FFM, along with deficits in bone and protein mass [24]. Adjusting for height, regional analyses showed substantial loss of FM in both limbs and trunk, whereas for FFM the main loss was in the limbs only. Other studies of adult women with anorexia nervosa using magnetic resonance imaging have shown that intra-abdominal fat is relatively preserved during weight loss and increases most during refeeding [37, 38]. These studies clearly show that severe weight loss affects all body components, but the results have limited significance for younger LMIC populations in which undernutrition is often associated with infection.

Given the high mortality risk associated with severe-acute undernutrition in LMICs, body composition measurements have historically been rare, but the emerging data show some patterns. Table 1 summarises the details of studies considered below, to illustrate how this field has progressed.

**Table 1:** Studies associating severe-acute undernutrition with body composition in children from low and middle-income countries

Country [Reference]	Location	Categories of undernutrition	Sample size	Age	BC techniques
Ethiopia [39]	Jimma (urban area)	Oedematous severe-acute undernutrition Non-oedematous severe-acute undernutrition Community controls	214 136 120	L (during treatment), 0–14 years at baseline	BIVA
DR Congo [14]	Kabare administrative zone, South Kivu province	MUAC < 115 mm or bilateral pitting oedema	47 controls, 55 cases	L (during treatment), 15 months (range 6–23 months) at baseline	BIVA, D_2_O
Malawi [16]	Blantyre	Severe-acute undernutrition survivors Sibling controls Community controls	352 184 217	L, median 9.3 years at follow-up (IQR 8–10)	BIVA, Grip strength
Cambodia [35]	Rural municipalities in Prey Veng province, southeast of Phnom Penh	WHZ < –2 WHZ −2 to −1 WHZ − 1 to 0 WHZ > 0	34 119 169 90	L, measurements at 6 and 15 months	D_2_O
Turkey [40]	Diyarbakir	‘Severe undernutrition’ assessed by WAZ	74 cases 47 controls	6–52 months	Ultra-sonography
Egypt [41]	Cairo	WAZ < 60%	30 cases 10 controls	6–36 months	Echo-cardiography
Thailand [42]	Chiang Mai	WHZ < 70%	11	10–43 months	Chest radiograph

Abbreviations: *BC* body composition, *BIVA* bioelectrical impedance vector analysis, *D*_*2*_*O* deuterium dilution, *DR* Democratic Republic, *IQR* interquartile range, *L* longitudinal study, *MUAC* mid-upper arm circumference*, WAZ* weight-for-age z-score, *WHZ* weight-for-height z-score

A recent study of children with severe-acute undernutrition in Ethiopia used a version of BIA known as bioelectrical impedance vector analysis (BIVA) to differentiate between low cell mass and dehydration/oedema. Consistent with simple clinical assessments, BIVA measurements successfully differentiated between dehydrated and oedematous children with severe-acute undernutrition [39]. Although BIVA does not give absolute values, both oedematous and non-oedematous children with severe-acute undernutrition had markers of low cell mass, broadly equivalent to reduced FFM, relative to healthy control children from the same setting. Oedematous children lost excess fluid during treatment, while non-oedematous children appeared to gain small amounts of fat-free tissue [39]. This methodology holds promise for incorporating assessment of hydration status in future studies. Moreover, BIVA parameters correlate with biochemical markers of nutritional status [43].

**Fig. 1:**
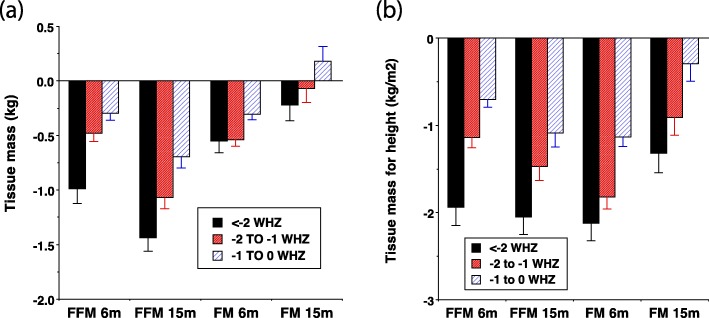
Association between body composition outcomes and the severity of wasting, categorised by weight-for-height z-scores. Data shown are from a longitudinal study of child undernutrition in rural Cambodia [35]. Effects for the three categories of low weight-for-height z-score (WHZ) are expressed relative to a reference group with WHZ > 0. **a** Absolute fat-free mass (FFM) and fat mass (FM). **b** Height adjusted fat-free mass index (FFMI) and fat mass index (FMI). Error bars are standard error of the mean

A study from Cambodia analysed data from a randomised trial investigating the potential of four ready-to-use therapeutic foods (RUTFs) to prevent undernutrition [44]. In this study, body composition was assessed using D_2_O at 6 and 15 months of age [35]. In observational analyses of these data, FFM declined strongly in proportion with the degree of wasting at both time-points, whereas declines in fat mass were more modest (Fig. 1). These contrasts broadly persisted following adjustment for height (i.e. analysing FFMI and FMI). The FFM deficits of wasted children increased with age, whereas FM deficits reduced [35], indicating a relative preservation of FM over FFM over time in severe undernutrition.

A long-term follow-up of survivors of severe-acute undernutrition in Malawi showed that, compared to community and sibling controls, the children had recovered some of their deficit in height, but nevertheless remained shorter in height with shorter leg lengths. They had smaller calf circumference and MUAC. BIVA revealed markers of lower FFM and they also had lower grip strength. They had similar waist girth, but lower hip girth, suggesting a more central distribution of body fat [16]. Although no direct body composition data were collected in this study, the available data indicate that the severe-acute undernutrition survivors had lower levels and functional capacities of FFM, alongside depleted peripheral but preserved central adiposity.

Very few studies have assessed the size of specific organs, but studies from Egypt and Thailand found that infants and young children with severe-acute undernutrition, whether oedematous or non-oedematous, had small heart volume and reduced cardiac muscle [41, 42]. A similar study of young children with marasmus from Turkey reported lower kidney volumes [40]. A systematic review of the effects of undernutrition on immune function found consistent evidence of severe thymus atrophy [45].

Overall, severe wasting clearly affects both fat and fat-free tissues and although levels of fat may subsequently recover, levels of FFM may remain low in the longer-term. A subtler finding is the relative preservation of central compared to peripheral fat depots. However, there are as yet minimal data on individual organs and the long-term consequences of severe-acute undernutrition for body composition remain poorly understood.

### Body composition associated with chronic undernutrition

A substantially greater proportion of the world’s children are underweight and/or stunted than those that are severely wasted [1], and they have participated in a larger number of studies, some of them longitudinal. Table 2 summarises the locations, sample sizes and measurement techniques of the relevant studies reviewed below.

**Table 2:** Studies associating chronic undernutrition with body composition in children from low and middle-income countries

Country [Reference]	Location	Categories of undernutrition	Sample size	Age	BC techniques
Brazil [22]	Slums of Sao Paulo	HAZ < –2 HAZ > –2	13 29	CS, 11–15 years	DXA
Peru [46]	Lowland (Pampas de San Juan de Miraflores, Lima), and highland (rural communities of Santillana and Vinchos Districts of Ayacucho Region)	Height analysed on continuous basis	Lowland 201 Highland 160	CS, 3–8.5 years	Waist girth
Jamaica [47]	Poor neighbourhoods of Kingston	HAZ < –2 HAZ > –2	103 64	L, stunting assessed at < 2 years; follow-up at 7, 11 and 17 years	Skinfolds, girths
Cambodia [35]	Rural municipalities in Prey Veng province, southeast of Phnom Penh	HAZ < –2 HAZ − 2 to − 1 HAZ − 1 to 0 HAZ > 0	34 119 169 90	L, measurements at 6 and 15 months	D_2_O
Nepal [48]	Janakpur, Terai District	HAZ < –2 HAZ > –2	309 494	L, stunting assessed at 2 years; follow-up at 8 years	BIA, skinfolds, girths, kidney dimensions
India [49]	Areas of low socioeconomic status in Mumbai city, Maharashtra	HAZ < –2 HAZ > –2	330 330	CS, 2–4 years	Skinfolds, girths
Senegal [50]	Niakhar rural district	HAZ < –2 HAZ > –2	81 286	L, stunting assessed at 6–18 months; follow-up at 11 and 15 years	Skinfolds, girths
South Africa [51]	Township setting outside Potchefstroom, North-West Province	HAZ < –2 HAZ > –2	32 146	CS, 13–18 year range	Skinfolds, girths, densitometry
South Africa [21]	Birth-to-Twenty cohort, Soweto, Johannesburg	HAZ <-2, HAZ >-2	Total 140	L, Stunting assessed at 1 and 2 years; follow-up at 8 years	DXA

Abbreviations: *BC* body composition, *BIA* bioelectrical impedance analysis, *CS* cross-sectional study, *D*_*2*_*O* deuterium dilution, *DXA* dual-energy X-ray absorptiometry, *HAZ* height-for-age z-score, *L* longitudinal study. The south Africa study measured stunting at two time points in 140 children, but did not quantify the numbers stunted at either time point

**Fig. 2:**
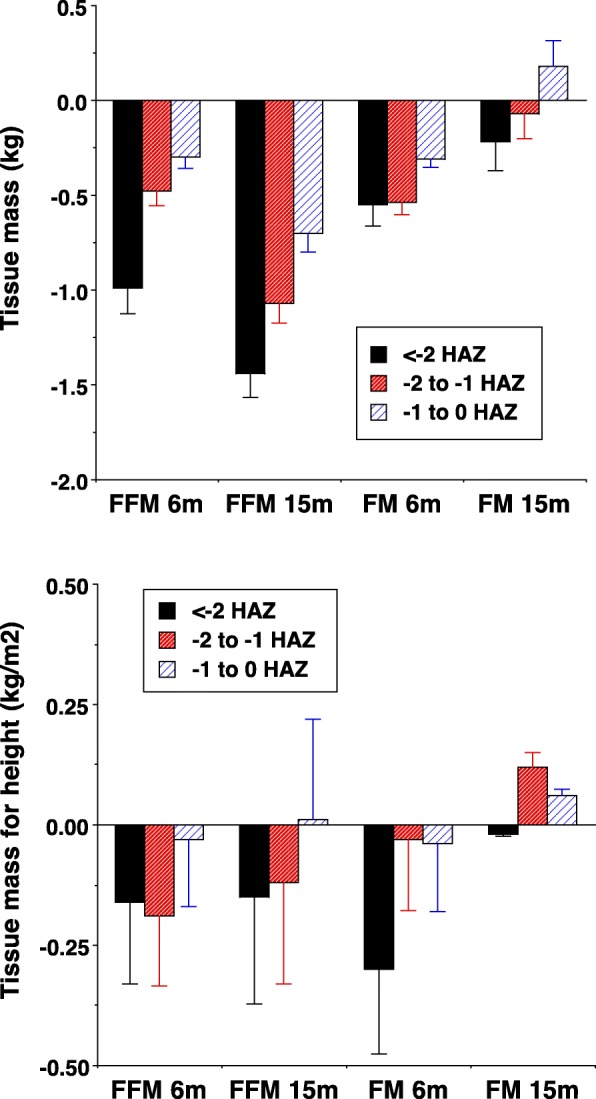
Association between body composition outcomes and the severity of stunting, categorised by height-for-age z-scores. Data shown are from a longitudinal study of child undernutrition in rural Cambodia [35]. Effects for the three categories of low height-for-age z-score (HAZ) are expressed relative to a reference group with HAZ > 0. **a** Absolute fat-free mass (FFM) and fat mass (FM). **b** Height adjusted fat-free mass index (FFMI) and fat mass index (FMI). Error bars are standard error of the mean

In the study from Cambodia discussed above, FFM declined strongly in proportion with the degree of linear growth retardation at both 6 and 15 months. However, these trends effectively disappeared on adjustment for height (i.e. analysing FFMI and FMI) (Fig. 2). Crude associations between FM and the magnitude of growth faltering were weaker; again they disappeared after adjusting for height [35]. In this study, therefore, tissue accretion was proportional to linear growth, with FFM most affected in absolute terms.

A study in Jamaica followed up children who had been either stunted or not at 2 years of age. Subcutaneous skinfold data was collected from participants at 17 years of age, as well as BMI data throughout adolescence. At 17 years old, previously stunted children remained shorter, had a lower BMI and showed no signs of greater central fat deposition. Instead, the stunted children showed faster linear growth between the ages of 3 and 11 years and had therefore reduced some of their height deficit by adolescence [47].

Similar findings were obtained in a follow-up of children in Nepal. Comparing children who had or had not been stunted at 2 years of age, those who had been stunted maintained a shorter height and had a lower BMI at 8 years of age [48]. FFM and kidney dimensions were reduced and these differences remained evident – albeit with smaller magnitude – after adjusting for their shorter height (i.e. analysing FFMI and FMI). The percentage deficit in FM at 8 years of age (~ 35%) was substantially greater than that in FFM (~ 15%). Both central and limb skinfold thicknesses were reduced, as were both waist and hip girths. There was no evidence of early-life stunting being associated with a more central fat deposition in this cohort.

However, other studies show contrasting patterns. Early studies of children from Brazilian shanty towns found that stunted adolescents, especially girls, had higher weight-for-height than healthy controls [52], suggesting an association between early linear growth faltering and later overweight. Subsequent studies using DXA found that stunted children gained less FFM than non-stunted children over a 3-year period, whereas boys, but not girls, gained more FM than controls [22]. Mechanistic studies in the same population found that stunted children had lower rates of fat oxidation than non-stunted children, which might predispose them to fat accretion [53]. Reduced fat oxidation in stunted children was also observed in children from North Korea [54]. However, a study in Cameroon did not replicate this association [55].

A longitudinal study of Senegalese girls found that those stunted at 6–18 months of age also had lower subcutaneous skinfold thickness, arm girth and BMI than their non-stunted peers at that time [50]. By the age of 15 years, the stunted girls had not caught up in height, but had caught up in body mass and no longer showed differences in arm girth or the sum of six skinfold thicknesses. However, there were subtle differences in fat distribution, with the stunted girls showing a slightly more central fat distribution in adolescence, as indicated by higher biceps and subscapular skinfolds.

A cross-sectional comparison of children living at high and low altitude in Peru (encompassing a range of height variation) found that the association between indices of adiposity and height varied markedly between the two settings [46]. At low altitude, height was positively associated with waist girth, suggesting that faster growing children were investing in both linear growth and adipose tissue deposition. However, at high altitude, height was inversely associated with waist girth, suggesting a trade-off between linear growth and fat deposition, with the shortest children investing relatively more in FM.

A cross-sectional study in India reported that stunted children aged 2–4 years had higher levels of total and central body fat than non-stunted counterparts [49]. In both sexes, stunted children were ~ 10 cm shorter than the non-stunted children. However, the ‘elevated body fat’ described by these authors in stunted children was not apparent in absolute terms and only emerged after adjusting for birthweight and change in weight z-score between birth and follow-up. Specifically, the authors’ statistical modelling tells us that if both stunted and non-stunted children had gained a similar amount of weight since birth, then it would be predicted that the stunted children would have greater adiposity. However, the stunted children had substantially lower postnatal weight gain than the non-stunted children. The shorter height and lower weight of the stunted children clearly indicates a substantial deficit in FFM. The purported increments in percentage fat and waist-for-height can be considered artefacts of this deficit, introduced by the statistical adjustments.

Similar concerns relate to a study of South African adolescents. Stunting was not associated with elevated skinfolds or waist girth. Although not significantly different, percentage fat was slightly higher among the stunted group and these individuals had a higher waist-to-hip ratio [51]. However, in recalculating the data, I revealed a substantial deficit in FFM in boys and a smaller effect in girls. Similarly, the higher waist-to-hip ratio in stunted adolescents can be attributed to low hip girth rather than high waist girth. This study did not, therefore, directly link stunting to elevated total or central adiposity. In a different cohort from Soweto, near Johannesburg, where body composition at 10 years of age was measured by DXA, stunting at 1 year of age was associated with lower FM and FFM at 10 years. At 2 years of age, stunting was associated only with lower FFM [21].

Overall, studies of stunted children have relatively consistently showed long-term deficits in FFM. In some cases, these deficits are proportional to the shorter height and in other cases the deficit persists even after adjusting for height. Whether stunting is causally associated with later adiposity remains less clear – an issue revisited below.

### Effects of treatment

A study in the Democratic Republic of Congo randomised young children with severe-acute undernutrition to different doses of RUTF. At the time of discharge, body composition was assessed using BIA and D_2_O, allowing the assessment of differences between patients and healthy controls and between the two trial groups. Compared with controls, the undernourished children still had lower FFM; however, the difference in FM was smaller and only significant in one of the two trial groups [14]. There were no significant differences in any body composition outcome between the two trial groups.

Regarding specific tissues and organs, an earlier study from Jamaica found that after clinical recovery from severe-acute undernutrition, infants’ muscle fibres increased in cross-sectional area, but remained small in size compared with controls. This suggests that complete muscle recovery is either slow or impossible [56]. However, the study of infants and young children with severe-acute undernutrition in Egypt reported a significant improvement in cardiac muscle following nutritional rehabilitation [41].

The reports of a potential association between stunting and fat accretion described above prompted concern that nutritional supplementation programmes, which deliberately seek to increase energy intake, might inadvertently elevate fatness in short children rather than promote growth in height and FFM. For example, earlier studies of nutritional treatment for severe-acute undernutrition reported high rates of fat accretion [57–59], which may be partly explained by diets lacking adequate micronutrients to support FFM accretion. Likewise, during recovery from long-term chronic undernutrition, adult participants in the 1950s Minnesota Starvation Study initially accumulated FM much faster than FFM, though baseline FFM was eventually recovered [60].

Of relevance here, the D_2_O method was recently used in a longitudinal trial in Burkina Faso to test the effects of 12 RUTFs in moderately undernourished young children [15]. This study had no control group, making it difficult to evaluate the deficits in FFM and FM prior to treatment, but – relative to UK reference data [28] – these children had major deficits in both tissues at baseline. Over 12 weeks, 93.5% of weight gain was accounted for by FFM. At the end of the study, FFM had increased by ~ 1 z-score and was similar to the UK reference median, whereas FM remained well below the UK median. Using height-adjusted outcomes, FFMI also increased by 0.80 kg/m^2^, whereas FMI showed a non-significant decline of 0.05 kg/m^2^. Stratifying children by height at baseline, there was no evidence that short children gained greater levels of FM during treatment [34].

In the similar trial from Cambodia, nutritional supplementation was provided for children with moderate-acute undernutrition for a longer period (6–15 months). In this trial, the average gain in FFM was 2.0 kg, while fat mass decreased, on average, by 0.2 kg, with no difference between the four trial groups receiving different RUTF formulations [44]. These two studies are consistent with other recent studies of treatment for severe-acute undernutrition [14, 61], in which, again, adiposity was not found to be elevated following RUTF treatment.

## Conclusions

Although further research is urgently needed, available data from LMICs on the association between undernutrition and body composition has revealed some relatively clear findings, as well as some areas of inconsistency.

First, all forms of undernutrition appear to adversely impact FFM, either in proportion to linear growth retardation or even more severely. These deficits often persist in the long-term and are associated with functional deficits, such as lower grip strength. However, a major gap in the literature relates to the effects of undernutrition on specific organs and muscle.

**Fig. 3:**
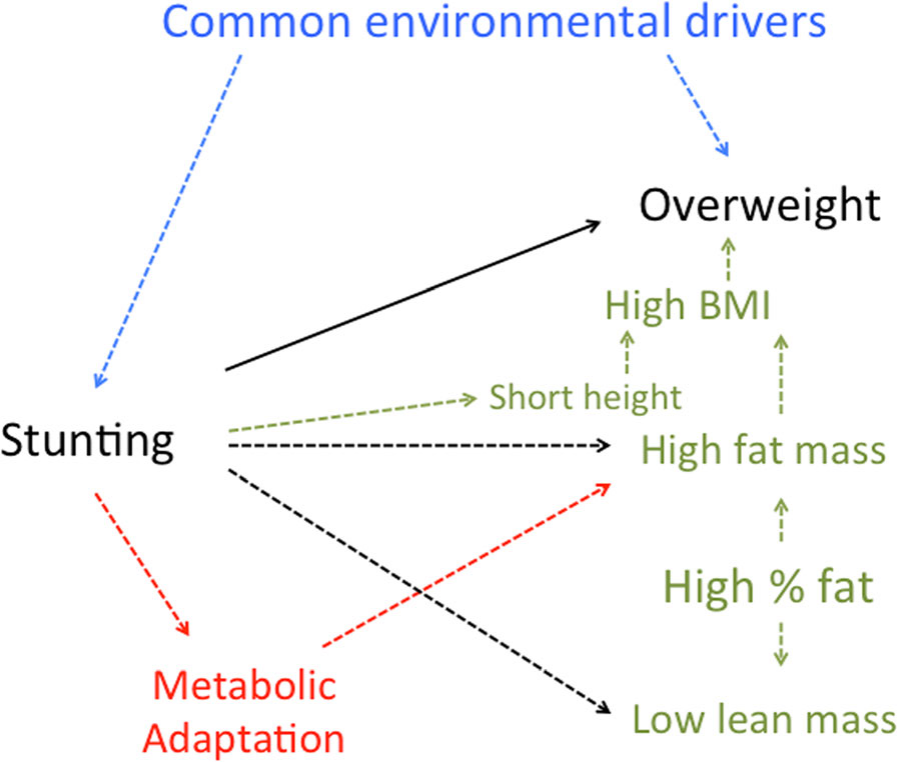
Possible pathways underlying the association between early-life stunting and subsequent body composition and nutritional status. Possible pathways may involve external environmental drivers (blue text), direct changes in growth and tissue masses (green text), or alterations in metabolic pathways (red text). The potential causality of this association requires further research

Second, undernutrition also reduces adiposity in the short-term. This is unsurprising, given that a key function of fat is to provide energy and molecular substrates for immune function when nutritional intake is depleted [10]. There are some indications that fat may be relatively preserved over time, suggesting that the body may sacrifice FFM to maintain crucial energy reserves for further ‘fire-fighting’. However, it is less clear whether children whose early growth was slowed are inherently at risk of gaining excess adiposity. Many studies fail to support this hypothesis and, while others might appear to support it, they suffer from statistical problems. Of those studies that did find a link between stunting and later elevated adiposity, the underlying mechanism remains unclear. Further research is needed to determine whether stunting alters metabolism or appetite in favour of fat accretion, or whether poor diets (energy dense but deficient in micronutrients) contribute to both stunting and overweight (Fig. 3). It is plausible that populations from different geographical regions have evolved contrasting biological responses to undernutrition; however, this hypothesis has yet to be explored.

Third, associations between undernutrition and body composition may contribute in the long-term to elevated non-communicable disease (NCD) risk. Low FFM indicates a low ‘metabolic capacity’ for homeostasis and thus a reduced ability to tolerate the ‘metabolic load’ associated with high levels of adiposity (regardless of how that adiposity accumulates) in later life [62, 63]. For example, short adult stature is a well-recognised risk factor for NCDs such as cardiovascular disease and diabetes [64, 65]. However, until adiposity becomes elevated, survivors of undernutrition may not present with overt NCD risk, as indicated by the lack of difference in risk markers compared to controls in the follow-up study in Malawi [16].

Finally, although methodological challenges remain, there is a steadily growing choice of methods appropriate for research on this topic, as well as more comprehensive reference data. As the agenda in child undernutrition moves beyond simply helping children to ‘survive’ in the short-term, to helping them ‘thrive’ in the long-term, body composition research will become increasingly important.

## Data Availability

Not applicable.

## Abbreviations

BIA: Bioelectrical impedance analysis
BIVA: Bioelectrical impedance vector analysis
BMI: Body mass index
D_2_O: Deuterium dilution
DXA: Dual-energy X-ray absorptiometry
FFM: Fat-free mass
FFMI: Fat-free mass index
FM: Fat mass
FMI: Fat mass index
HAZ: Height-for-age z-score
LMIC: Low- and middle-income countries
MCM: Multi-component model
MUAC: Mid-upper arm circumference
NCD: Non-communicable disease
RUTF: Ready-to-use therapeutic food
WHZ: Weight-for-height z-score
